# Complex transmission of partiti-, ambi- and ourmiaviruses in the forest pathogen *Heterobasidion parviporum*

**DOI:** 10.1016/j.virusres.2024.199466

**Published:** 2024-10-09

**Authors:** Muhammad Kashif, Anna Poimala, Eeva J. Vainio, Suvi Sutela, Tuula Piri, László Benedek Dálya, Jarkko Hantula

**Affiliations:** a Natural Resources Institute Finland (Luke), Latokartanonkaari 9, FI‐00790 Helsinki, Finland; b Mendel University of Agriculture and Forestry, Brno, Czech Republic

**Keywords:** *Heterobasidion*, Mycovirus, Partitivirus, Transmission, Ourmiavirus, Ambivirus, Coinfection, Dual culture

## Abstract

Utilizing Heterobasidion partitivirus 13 strain an1 (HetPV13-an1) and 15 strain pa1 (HetPV15-pa1) in co-infection is considered a potential biocontrol approach against Heterobasidion root and butt rot. Both partitiviruses mediate debilitating effects in most *Heterobasidion* host isolates and are generally transmitted efficiently between host strains. In this investigation, we conducted transmission experiments in the laboratory (*in vitro*) using several *H. parviporum* isolates to test whether using dual partitivirus infections is a more efficient way of transmitting viruses to new hosts compared to using single partitivirus infections, and whether co-occurring single-stranded RNA (ssRNA) viruses are co-transmitted during the process. The results showed that *H. parviporum* donors carrying both partitiviruses, HetPV13-an1 and HetPV15-pa1, transmitted HetPV15-pa1 more efficiently to recipients than the same donors infected with only HetPV15-pa1. In contrast, the transmission of HetPV13-an1 did not differ significantly between donors infected with both or only one partitivirus. Altogether, the transmission rates of HetPV13-an1 and HetPV15-pa1 were high on artificial media. Moreover, the transmission of the ssRNA viruses Heterobasidion ourmia-like virus 1(HetOlV1-pa7) and 4 (HetOlV4-an1) as well as Heterobasidion ambi-like virus 3 (HetAlV3-pa4) across different recipients were found to be variable. This study demonstrated for the first time the transmission of ambi- and ourmiaviruses between *H. parviporum* isolates in dual cultures and showed that *H. parviporum* mycelia can be cured of these ssRNA viruses using heat treatment.

## Highlights

• The intraspecies transmission rate of partitiviruses is high *in vitro*.
• Double- and single-partitivirus-infected donors transmit partitiviruses alike.
• In co-infections partiti- and ssRNA viruses affect each other's transmission.
• Ambi- and ourmiaviruses are transmitted between conspecific *H. parviporum*.

## 1 Introduction

Fungal pathogens of the genus *Heterobasidion* cause root and butt rot in conifers of boreal and temperate forests. Two different species, *H. annosum* and *H. parviporum*, are native to Fennoscandia and have preferences for infecting pines and spruces, respectively (Niemelä and Korhonen, 1998; Gonthier and Garbelotto, 2011). *Heterobasidion* spp. are efficiently disseminated via basidiospores, causing primary infections when spores land on fresh stumps and wounds. Subsequently, the fungal infection spreads vegetatively to the root systems of trees and, ultimately, to neighboring trees, forming disease centers that may persist for centuries (Korhonen, 1978; Redfern and Stenlid, 1998; Woodward et al., 1998).

The *H. annosum* s. lat. cluster includes three European species as *H. parviporum, H. annosum* and *H. abietinum* (Niemelä and Korhonen 1998). *H. annosum* s.s. and *H. parviporum* have preferences to cause infection in pines and Norway spruce, respectively (Korhonen and Piri, 1994; Korhonen et al. 1998). In Finland, economic losses caused by *H. parviporum* surpass those caused by any other biotic or abiotic agents (Hantula et al., 2023). In addition to direct damage, *H. parviporum*-infected spruces are susceptible to wind damage, which can be followed by bark beetle attacks and subsequently lead to forest fires (Oliva et al., 2011; Honkaniemi et al., 2017; Nevalainen, 2017). These fungi thrive in managed forests, and it is anticipated that predicted climatic change will amplify the damage caused by them (Müller et al., 2014). Consequently, new methods are needed to control the spread of the disease at forest sites where it is already present. One of these methods being considered is the use of fungal viruses (Vainio and Hantula, 2016).

Most mycoviruses have double-stranded RNA (dsRNA) or single-stranded RNA (ssRNA) genomes (Kondo et al., 2022; Ayllón and Vainio, 2023; Sato and Suzuki, 2023). They are ubiquitously present in species of fungi but usually lack extracellular infective particles, and therefore disperse only intracellularly via anastomoses or through sexual or asexual spores (Hillman and Milgroom, 2021; Ayllon and Vainio, 2023; Hough et al., 2023). Although most mycoviruses do not mediate obvious host symptoms but remain cryptic, there are also viruses causing debilitating or hypovirulence effects on their fungal hosts (Day et al., 1977; Ahn and Lee, 2001; Osaki et al., 2002; Yu et al., 2010; Ghabrial et al., 2015; Vainio et al., 2018; van Diepeningen 2021; Hough et al., 2023).

RNA viruses are commonly found and taxonomically diverse in isolates of *Heterobasidion*. The observed viruses belong to various families, including *Partitiviridae, Curvulaviridae, Mitoviridae, Narnaviridae, Botourmiaviridae, Dumbiviridae* and *Trimbiviridae* (Vainio and Hantula, 2016; Vainio et al., 2018; Sutela et al., 2021; Kuhn et al., 2024; Dálya et al., 2024)*.* Two *Heterobasidion* partitiviruses, HetPV13-an1 and HetPV15-pa1, have been demonstrated to have highly negative phenotypic effects on certain *Heterobasidion* strains and are being tested as potential biocontrol agents (Vainio et al., 2018; Kashif et al., 2019). Environmental conditions have also been shown to impact virus-fungus relationships (Hyder et al., 2013; Jurvansuu et al., 2014). The transmission abilities, phenotypic effects, and interactions of ssRNA viruses with dsRNA viruses during co-infection are poorly known in *Heterobasidion*. As these interactions might affect the biocontrol abilities of partitiviruses, examination of the virus-virus and virus-fungus relationships in *Heterobasidion* fungi is crucial for developing an effective practical control application.

In this investigation we aimed to determine whether coinfecting partitiviruses HetPV13-an1 and HetPV15-pa1 enhance each other's transmission rates between *H. parviporum* isolates. We also studied if Heterobasidion ourmia- and ambiviruses are transmitted through anastomosis between interacting *H. parviporum* mycelial isolates, and whether debilitating partitiviruses HetPV13-pa1 and HetPV15-pa1 affect the transmission rate of co-infecting ssRNA viruses. The curing of *H. parviporum* isolates of ssRNA viruses using heat treatments was also examined.

## 2 Materials and methods

### 2.1 Preparation of donor and recipient strains

**Fig. 1:**
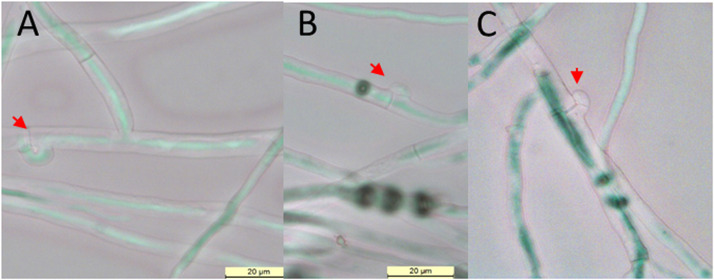
Loop shaped structure known as clamp connection, a distinctive feature of heterokaryotic *Heterobasidion* fungi, in isolates SB9.3 (A-B) and KS92 (C). Clamps in the mycelium are shown by red arrows.

Prior to setting up virus transmission experiments, the *H. parviporum* isolates used as virus donors or recipients were examined under microscope and confirmed to be heterokaryotic based on the presence of clamp connections (Fig. 1). By using a heterokaryotic donor and recipient we aimed to exclude the possibility of mating between the co-cultured isolates leading to changing of their nuclear status (*i.e.*, two homokaryons creating a heterokaryon or nuclear transmission from a heterokaryon to a homokaryon, also called Buller phenomenon), which would not be indicative of virus transmission potential between somatically incompatible host strains (Korhonen, 1978).

The process of preparing virus-free *H. parviporum* isolates as recipients for *in vitro* transfer experiments under laboratory conditions began with RNA-sequencing to identify any existing RNA virus infections and transcripts of DNA viruses (Vainio et al., 2015a; Sutela et al., 2021). Both fungal strains (SB6.26 and SB9.3) constructed to be used as virus donors were also subjected to RNA-seq (Table 1, Supplementary table S1-S2). Both of the original isolates had been collected from Solböle in 2005 by Tuula Piri.

**Table 1:** *Heterobasidion parviporum* isolates used as virus donors in the current study.

Isolate name	Viruses of the isolate	GenBank accession	RNA-Seq pool
SB6.26-PV13-15-OlV4-AlV3	HetPV13-an1	KF963177^1^	PhyHet
	HetPV15-pa1	KF963186^1^	
	HetOlV4-an1^2^	OR083038	
	HetAlV3-pa4^3^	OR051024^3^	
SB6.26-PV13-OlV4-AlV3	HetPV13-an1	KF963177^1^	
	HetOlV4-an1	OR083038	
	HetAlV3-pa4^3^	OR051024^3^	
SB6.26-PV15-OlV1-OlV4-AlV3	HetPV15-pa1	KF963186^2^	
	HetOlV1-pa7,	OR051025	
	HetAlV3-pa4^3^	OR051024^3^	
SB9.3-PV13-15-OlV1-OlV4-AlV3	HetPV13-an1	KF963177^1^	PhyHet
	HetPV15-pa1	KF963186^1^	
	HetOlV1-pa7	OR051025	
	HetOlV4-an1	OR083038	
	HetAlV3-pa4^3^	OR051023^3^	
SB9.3-PV15-OlV1-OlV4-AlV3	HetPV15-pa1	KF963186^1^	
	HetOlV1-pa7	OR051025	
	HetOlV4-an1	OR083038	
	HetAlV3-pa4^3^	OR051023^3^	
SB9.3-PV13-OlV4-AlV3	HetPV13-an1	KF963177^1^	
	HetOlV4-an1	OR083038	
	HetAlV3-pa4^3^	OR051023^3^	

The principle of naming host strains harboring viruses strains follows that of Hantula et al. (2020); HetPV=Heterobasidion partitivirus, HetOlV=Heterobasidion ourmia-like virus, HetAlV= Heterobasidion ambi-like virus, HetRV=Heterobasidion RNA virus. HetOlV4-an1 has also been referred to as Heterobasidion ourmia-like virus 4 strain pa2 in the submitted sequence data (sequence accession files) in NCBI Genbank.

1 virus sequence determined previously by Kashif et al. (2015).

2 There are two variants of HetOlV4-an1, the other reported in Roininen et al. 2024.

3 virus strain HetAIV3-pa4 has been designated into two variants a and b originated from SB6.26-PV13–15-OlV4-AlV3 and SB9.3-PV13–15-OlV1-OlV4-AlV3 host isolates, respectively and differ based on SNPs.

### 2.2 RNA sequencing

Fungal samples were processed for RNA sequencing (RNA-seq) to detect unkown RNA viruses. The fungal strains were incubated on modified orange serum (MOS) agar plates at +20 °C for 14 days, after which mycelium was collected in Falcon tubes. The mycelium was freeze-dried for one to two days and stored at −80 °C. Homogenization of samples was done with FastPrep-24™ (MP Biomedicals, Santa Ana, CA, USA) using 1–2-mm quartz sand grains and RNA was extracted using Spectrum Plant Total RNA Kit (Sigma) according to manufacturer's instructions. The quantity of RNA was measured using a NanoDrop™ One^C^ spectrophotometer (Thermo Fisher Scientific) and its quality was assessed on an agarose gel, and thereafter the samples were divided into two pooled libraries of 6 and 11 samples, each containing 1 μg of RNA from each isolate. The RNA-Seq libraries containing each sample are indicated in Table 1, Table 2. Deeper quality check of RNA, library construction and sequencing were conducted at Macrogen Europe. The RNA integrity numbers (RINs) of libraries Kashif and PhyHet were 7.0 and 10.0 respectively. The libraries were constructed with TruSeq Stranded Total RNA with Ribo-Zero H/M/R Gold (Illumina) and sequencing was performed using an Illumina NovaSeq 6000 system generating stranded paired-end sequences.

**Table 2:** Cured^1^*Heterobasidion parviporum* isolates used as virus recipients in the current study.

Isolate name	Viruses in the original non-cured host	GenBank accession (coding complete)	Origin of isolate and year of collection or reference^3^
RK5A	HetAlV10-pa1, HetAlV12-pa1	OR644498	Ruotsinkylä, 2010, Eeva Vainio & Tuula Piri
		OR644499	Vainio et al., 2013
SB2011	HetPV13-an1, HetAlV1-pa2, HetAlV3-pa3, HetAlV10-pa1, HetAlV12-pa1	KF963177^2^	Solböle, 2011, Tuula Piri
		PP951620	
		OR644497	
		OR644498	
		OR644499	
LAP3.3.11	HetAlV10-pa1	OR644498	Lapinjärvi, 1991, Tuula Piri
KS92	HetPV13-an1, HetRV6-pa36, HetAlV1-pa2, HetAlV3-pa3, HetAlV10-pa1, HetAlV12-pa1, HetpaFV1-pa1	KF963177^2^	Karkkila 2017, Jarkko Hantula
		OR644495	
		PP951620	
		OR644497	
		OR644498	
		OR644499	
		OR644501	

The principle of naming host strains harboring viruses follows that of Hantula et al. (2020); HetPV=Heterobasidion partitivirus, HetAlV= Heterobasidion ambi-like virus, HetRV=Heterobasidion RNA virus, HetpaFVl=Heterobasidion parviporum fusarivirus 1.

1 The *H. parviporum* isolates were heat-treated in order to cure them from virus infections observed prior to heat-treatments with RNA-sequencing (RNA-Seq pool Kashif) and RT-PCR.

2 virus sequence determined previously by Kashif et al. (2015)

Bioinformatic analysis of RNA-sequencing data followed the pipeline of Sutela et al. (2021). The reads were cleaned using Trimmomatic and de novo assembled with Trinity (v. 2.8.5). The assembled contigs were compared to a custom viral sequence database (obtained from Genbank by downloading *Riboviria, Genomoviridae*, and unclassified virus protein sequences after excluding the most abundant (human/animal) virus sequences and nucleocytoplasmic large DNA viruses) with BLASTx to search for contigs having homology with virus sequences. Trinity contigs with similarity to predicted host proteins (*H. irregulare,* Olson et al. 2012) were depleted *in silico*. Virus-like sequences detected in each RNA-Seq library were allocated to specific *H. parviporum* isolates by RT-PCR using virus specific primers (Table S1). The level of sequence variation was also examined by mapping of raw RNA-Seq reads against Trinity-assembled contigs. The complete genomes of the circular ambiviruses detected in the host isolates (SB9.3 and SB6.26) were determined by Sanger sequencing (primers given in Table S1).

### 2.3 Heat treatment of fungal isolates

Heat treatments were applied to cure the recipient isolates (Table 2) of pre-existing viruses. The four *H. parviporum* isolates of the RNA-Seq pool Kashif were subjected to heat-treatments: two days old *H. parviporum* mycelium on 2 % malt extract agar (MEA) was incubated at +30 °C for three days and thereafter for five days at each of +32 °C and +34 °C, respectively, followed by a recovery of three days at +20 °C (Vainio et al., 2018; Kashif et al., 2019), after which an inoculum from the treated mycelium was transferred to a new MEA media plates (3–5 subcultures) for further recovery of at least 5–7 days. All the heat-treated subcultures were found virus-free based on RT-PCR using virus specific primers (Supplementary Table 1).

### 2.4 *In vitro* transmission experiments

Two donor strains, SB9.3 and SB6.26 (Table 1), with three different partitivirus statuses (HetPV13-an1, HetPV15-pa1 or coinfection of HetPV13-an1 and HetPV15-pa1) were selected to transmit viruses to the four virus-free recipients (RK5A, SB2011, LAP3.1.1, and KS92) (Table 2). The transmission experiment was conducted using 20 dual cultures prepared on MEA as previously described (Vainio et al., 2013; Kashif et al., 2019). The total number of transmission trials was 420. Each agar plate was incubated at +20 °C for 60 days, and thereafter subcultured by transferring an inoculum from the recipient side as a 0.5 × 0.5 cm piece of MEA containing actively growing mycelia taken about 1 cm from the edge of the culture.

After the incubation, virus transmissions into recipients were examined by RNA extraction and cDNA synthesis, followed by RT-PCR. Total RNA from fungal mycelia of *H. parviporum* isolates was extracted using the TRI Reagent (Molecular Research Center Inc., USA) method. Mycelium was grown for 1–2 weeks followed by homogenization with 1–2 mm quartz sand in TRI Reagent using Fast-Prep F (Jurvansuu et al., 2014). cDNA was synthesized using 2 μg of total nucleic acids and RevertAid Reverse Transcriptase (Thermo Fisher) according to manufacturer´s instructions, followed by detection of transmitted viruses by RT-PCR using virus specific primers (Supplementary Table 1) and DreamTaq DNA Polymerase (Thermo Fisher). PCR products were detected with agarose gel electrophoresis and visualized by UV light. Positive subcultures (3–5) were purified from the very edge of the 2–4 days old mycelium. The stability/presence of the viruses in the donor strains was confirmed by RT-PCR using specific primers. The presence of viruses was validated as described above by RT-PCR. Pairing tests were made for the virus hosting subcultures against donor and original recipient strains to confirm the genotype of the new host after dual culture (Stenlid, 1985).

The statistical significance of the difference between transmissions rates from hosts with different virus statuses was tested using Fisher's exact test.

## 3 Results

### 3.1 Viruses detected by RNA-Seq

RNA-sequencing (RNA-seq) was conducted to detect virus infections in the *H. parviporum* strains (Table 1, Table 2). The analysis showed that they were always infected by one or more viruses before heat treatments. Strains of known partitiviruses and new ourmia- and ambiviruses as well as a novel fusarivirus, named here as Heterobasidion parviporum fusarivirus 1 (HetpaFV1), were observed among the analyzed *Heterobasidion* isolates. GenBank accession numbers for coding complete sequences of the newly detected viruses and variants are reported in Table 1, Table 2. The sequencing data was compared to a custom viral database based on BLASTx analysis (NCBI) and viruses retrieved with some filtering of most abundant viral sequences (Supplementary Table S3).

However, more detailed phylogenetic analysis is beyond the scope of this study. Schematic representation of the genome organization of the novel fusarivirus, HetpaFV1 is given in Supplementary Figure S1.

The donor SB6.26 hosted a novel ourmia-like virus, designated here as Heterobasidion ourmia-like virus 4 (HetOlV4-an1), this virus strain appeared to be originated from other host isolate 94233 as well as a new strain of Heterobasidion ambi-like virus 3 (named HetAlV3-pa4; Sutela et al., 2021). In addition to these two viruses, donor SB9.3 also hosted a new strain of Heterobasidion ourmia-like virus 1 (named HetOlV1-pa7; Sutela et al., 2021). The coding complete sequences of HetOlV1-pa7 and HetOlV4-an1 as well as the complete genome sequences of the HetAlV3 variants from hosts SB9.3 and SB6.26 were submitted to Genbank and accessions are given in Table 1.

### 3.2 Curing *H. parviporum* isolates of viruses

*H. parviporum* isolates were cured of both dsRNA and ssRNA viruses during the heat treatments (Table 2). After the heat treatment all *H. parviporum* isolates (to be used here as virus transfer recipients) were confirmed by RT-PCR to be virus free.

### 3.3 Viral transmission between *H. parviporum* isolates

Transmission experiments were conducted using two host genotypes of *H. parviporum* (SB6.26 and SB9.3) as donors. The overall transmission rates were generally high for partitiviruses (ranging between 69 % and 100 %), but considerably more variable for the ssRNA viruses (ranging between 0 % to 100 %) (Table 3). Statistical tests were conducted for cases where only one variable (virus content or host strain) differed between the compared isolates.

**Table 3:** Transmission frequencies (%) of HetPV13-an1 and HetPV15-pa1 viruses from *H. parviporum* donors hosting one or two partitiviruses to virus-free recipients of the same species.

Donor	SB6.26-PV13-15-OlV4-AlV3		SB6.26-PV13-OlV4-AlV3	SB6.26-PV15 -OlV1-OlV4-AlV3	SB9.3-PV13-15-OlV1-OlV4-AlV3		SB9.3-PV13 -OlV4-AlV3	SB9.3-PV15 -OlV1-OlV4-AlV3
Virus/Recipient	HetPV13-an1	HetPV15-pa1	HetPV13-an1	HetPV15-pa1	HetPV13-an1	HetPV15-pa1	HetPV13-an1	HetPV15-pa1
SB2011	70 % (14/20)	95 % (19/20)	85 % (17/20)	90 % (18/20)	69 % (11/16)	88 % (14/16)	85 % (17/20)	70 % (14/20)
RK5A	100 % (20/20)	100 % (20/20)	90 % (18/20)	100 % (20/20)	79 % (15/19)	95 % (18/19)*	100 % (20/20)	70 % (14/20)*
KS92	100 % (19/19)	100 % (19/19)*	95 % (19/20)	21 % (4/19)*	100 % (20/20)	100 % (20/20)	100 % (20/20)	100 % (18/18)
LAP3.3.11	(ND)	(ND)	(ND)	(ND)	88 % (15/17)	71 % (12/17)	100 % (19/19)	70 % (14/20)

(ND) refers to isolates that were not determined as the recipient strain was not used for the transmission from donor SB6.26.

⁎ P-value threshold: = ≤ 0.05.

Using this principle, we observed, using strains hosting one (HetPV13-an1) or two partitiviruses (HetPV13-an1+HetPV15-pa1) in addition to HetOlV4 and HetAlV3, that coinfecting HetPV15 in the donor SB6.26 did not affect the transmission efficacy of HetPV13-an1 (Table 3). Also, the differences in transmission frequency of HetPV13-an1 from the two donor genotypes (SB6.26 and SB9.3) and between the three recipients (RK5A, SB2011 and KS92) were nonsignificant (Table 3, Table S4).

Comparisons could also be made on the effect of viral transmission from experiments conducted using strains of genotype SB9.3 hosting either one (HetPV15-pa1) or two partitiviruses (HetPV13-an1 and HetPV15-pa1), as well as HetOlV4 and HetAlV3. The coinfecting partitivirus HetPV13-an1 significantly increased transmission of HetPV15-pa1 to one (RK5A) out of the four recipients (Table 3, Table S4). The partitivirus infection status of the donor infected with two ssRNA viruses did not affect the transmission frequency of HetOlV4-an1 or HetAlV3-pa4 (Table 4 a). The double partitivirus infection (HetPV13-an1 and HetPV15-pa1) in the donor significantly increased transmission of HetOlV1-pa7 to two recipients (RK5A and KS92) but decreased it to another one (LAP3.3.11) and had no effect in the transmission to the fourth recipient (Table 4 b). The coinfecting HetPV13-an1 almost completely inhibited transmission of HetOlV4-an1 to one of the recipients (LAP3.3.11) but had insignificant effect on three others. Also, coinfecting HetPV13-an1 reduced transmission of HetAlV3-pa4 to two of the recipients (RK5A and LAP3.3.11) but increased transmission to the third (KS92) and had no effect to the fourth recipient (Table 4 b, Table S5).

**Table 4:** a) Transmission frequencies (%) of ssRNA viruses from two *H. parviporum* donors with double (SB9.3-PV13-15-OlV1-4-AlV3) or single (SB9.3-PV15-OlV1-4-AlV3) partitivirus infection to virus-free recipients of the same species. (a) transmission of ssRNA viruses from donors infected with two ssRNA viruses (HetOlV4-an1 and HetAlV3-pa4). (b) transmission of ssRNA viruses from donors infected with three ssRNA viruses (HetOlV1-pa7, HetOlV4-an1 and HetAlV3-pa4a).

a)						
Virus	HetOlV4-an1			HetAlV3-pa4		
Donor/Recipient	SB9.3-PV13-OlV4-AlV3	SB6.26-PV13-OlV4-AlV3	SB6.26-PV13-15-OlV4-AlV3	SB9.3-PV13-OlV4-AlV3	SB6.26-PV13-OlV4-AlV3	SB6.26-PV13-15-OlV4-AlV3
RK5A	95 % (19/20)	95 % (19/20)	95 % (19/20)	80 % (16/20)*	100 % (20/20)	100 % (20/20)
SB2011	80 % (16/20)	100 % (20/20)	95 % (19/20)	55 %⁎⁎(11/20)	100 % ⁎⁎ (20/20)	95 % (19/20)
LAP3.3.11	5 % (1/20)	(ND)	(ND)	100 % (20/20)	(ND)	(ND)
KS92	100 % (20/20)	(ND)	(ND)	90 % (18/20)	(ND)	(ND)
*Average*	70	(ND)	(ND)	78	(ND)	(ND)

b)						
Virus	HetOlV1-pa7		HetOlV4-an1		HetAlV3-pa4	
Donor/Recipient	SB9.3-PV15- OlV1-4-AlV3	SB9.3-PV13-15- OlV1-4-AlV3	SB9.3-PV15- OlV1-4-AlV3	SB9.3-PV13-15- OlV1-4-AlV3	SB9.3-PV15- OlV1-4-AlV3	SB9.3-PV13-15-OlV1-4-AlV3
RK5A	50 %* (10/20)	85 %* (17/20)	10 % (2/20)	10 % (2/20)	100 %⁎⁎ (20/20)	50 %⁎⁎ (10/20)
SB2011	60 % (12/20)	82 % (14/17)	5 % (1/20)	0 % (0/20)	60 % (12/20)	71 % (12/17)
LAP3.3.11	100 %* (20/20)	76 %* (13/17)	100 %⁎⁎ (20/20)	0 %⁎⁎ (0/20)	95 %⁎⁎ (19/20)	29 %⁎⁎ (5/17)
KS92	39 %⁎⁎ (7/18)	100 %⁎⁎ (18/18)	11 % (2/18)	0 % (0/20)	11 %⁎⁎ (2/18)	94 %⁎⁎ (17/18)
*Average*	63	86	31	3	68	61

(ND) refers to isolates that were not determined as the recipients for the transmission from donor SB6.26.

⁎⁎ P-value thresholds: = ≤ 0.005.

⁎ = ≤ 0.05.

## 4 Discussion

In our previous experiments, partitiviruses HetPV13-an1 and HetPV15-pa1 enhanced each other´s transmission rates between the *H. annosum* isolates used (Kashif et al., 2019). In this investigation we tested whether this phenomenon is generalizable to *H. parviporum* using a larger collection of isolates under laboratory conditions. Our results from transmission experiments, where we considered only differences between donors with identical virus contents, showed that the relationship between double-infecting partitiviruses was considerably more complicated than expected. When present in a mixed infection, HetPV13-an1 and HetPV15-pa1 significantly enhanced each other´s transmission rates between two isolate pairs of *H. parviporum* under laboratory conditions. In one isolate pair, coinfection enhanced the transmission of HetPV13, and in the case of another isolate pair, HetPV15 transmission was enhanced by double-infection. The transmission of single and double infections of the same viruses HetPV13-an1 and HetPV15-pa1 in *H. annosum* was conducted by Roininen et al. (2024), but in those experiments the double infections did not increase the transmission efficacy.

Overall partitiviruses’ transmission from both double infected donors showed that both virus strains HetPV13-an1 or HetPV15-pa1 transmitted to the recipient isolates. However, the transmission frequencies were not found statistically higher when compared to donors infected with only one partitivirus (HetPV13-an1 or HetPV15-pa1). Therefore, we may conclude that our studies (based on Kashif et al., 2019) on the generally positive effect of HetPV13-an1 and HetPV15-pa1 on each other´s transmission under laboratory conditions was not well supported. It should, however, be noted that in view of a biocontrol application, a double infected treatment strain (biocontrol agent) may be feasible as the transmission rate of at least one partitivirus was high in most cases. These findings accord with previous studies showing high virus transmission rates on artificial media (Ihrmark et al., 2002; Vainio et al., 2010; 2013; 2015b; 2017; Kashif et al., 2019; Hantula et al., 2020) and, as such, are promising for the development of biocontrol applications. However, it should be noted that we are still lacking information about Heterobasidion partitivirus transmission rates among *H. parviporum* mycelia in living trees and also between different root systems of neighboring trees, both of which would be of high practical value. Furthermore, future studies should address the level of variation in host phenotypic response to dual infections by the debilitating viruses and to HetPV15-pa1 infection (*i.e.*, to what extent there is tolerance towards virus effects in the natural host population harboring also pre-existing viruses). For HetPV13-an1, there is a large variation in host response towards the virus (Vainio et al., 2018).

As *Heterobasidion* strains are known to be commonly infected by ssRNA viruses (Sutela et al., 2021), their presence was also considered in this investigation. Our aim was to use recipient isolates cured of viruses in order to avoid the possibility that pre-existing viruses in the recipient would hinder or increase viral transmission and thereby complicate the experimental setup. Earlier studies on *H. parviporum* support the notion that very closely viral variants may exclude each other from the host mycelium (Vainio et al., 2015b), while different partitivirus species are able to co-exist in multiple co-infection both naturally (Vainio et al., 2015) and when infected through contacting mycelia in the laboratory (Hantula et al., 2020).

First, we showed that, *H. parviporum* mycelia was successfully cured of ourmia- and ambiviruses by a heat treatment. Recipient isolates subjected to thermal treatment grew normally and vigorously and did not lose their ability for anastomoses as revealed by efficient virus receival (Vainio et al., 2018; Kashif et al., 2019). Secondly, we analyzed transmission of ambi- and ourmiaviruses and showed that both taxa may be transmitted between conspecific *H. parviporum* mycelia in dual cultures. This investigation is, to our knowledge, the first where ambiviruses are shown to be transmitted between two interacting *H. parviporum* strains, probably via anastomoses. This is also the first estimation of *in vitro* transmission frequency of ourmiaviruses in *H. parviporum*, and consistent with the findings of Sutela et al. (2021) who found that many *H. parviporum* strains hosted very closely related HetOlV1-pa5 variants in a single clone, which suggests efficient intramycelial transmission *in vivo*. In the ascomycete plant pathogen *Sclerotinia sclerotiorum,* ourmiaviruses have been previously shown to transmit readily via anastomosis (Wang et al., 2020). In addition, we showed that partitiviruses affected transmission rates of both ourmia- and ambiviruses, but different recipient strains showed contrasting responses (with increased or significantly decreased transmission efficiencies). Using naturally infected and isogenic cured strains as recipients would be an interesting topic for future studies. Finally, we observed a new fusarivirus during this investigation. That adds to our previous knowledge about the virome of *H. parviporum* and strengthens the view that this species hosts a highly diverse virus community (Dálya et al., 2024; Sutela et al., 2021).

As a conclusion, we showed that debilitating partitiviruses of *Heterobasidion* spp., HetPV13-an1 and HetPV15-pa1, as well as ssRNA viruses commonly observed in *Heterobasidion* mycelia affect each other´s transmission in a complicated way when coinfecting the same donor mycelium. Our investigation also showed that ambiviruses are able to be transmitted between contacting *H. parviporum* mycelia and added fusariviruses to the long list of viruses hosted by this species. Furthermore, the transmission rates of the two partitiviruses were found to be very high under laboratory conditions which suggest high potential for a biocontrol application. Future studies should investigate the transmission efficiency and effect of mycoviruses on the pathogen caused damage in living young trees suffering from Heterobasidion root rot.

## CRediT authorship contribution statement

**Muhammad Kashif:** Writing – review & editing, Writing – original draft, Visualization, Validation, Methodology, Investigation, Formal analysis, Data curation. **Anna Poimala:** Writing – review & editing, Writing – original draft, Visualization, Validation, Methodology, Investigation, Formal analysis, Data curation. **Eeva J. Vainio:** Writing – review & editing, Writing – original draft, Validation, Supervision, Resources, Methodology, Funding acquisition, Formal analysis, Conceptualization. **Suvi Sutela:** Writing – review & editing, Writing – original draft, Validation, Investigation, Formal analysis. **Tuula Piri:** Writing – review & editing, Writing – original draft, Supervision, Methodology, Formal analysis, Data curation. **László Benedek Dálya:** Writing – review & editing, Methodology, Investigation. **Jarkko Hantula:** Writing – review & editing, Writing – original draft, Validation, Supervision, Resources, Funding acquisition, Formal analysis, Data curation, Conceptualization.

## Declaration of competing interest

The authors declare that they have no known competing financial interests or personal relationships that could have appeared to influence the work reported in this paper.

## Data Availability

Data will be made available on request.

## Appendix Supplementary materials

Image, application 1

Image, application 2

## References

[bib0001] Ahn I.P., Lee Y.H. (2001). A viral double-stranded RNA up regulates the fungal virulence of *Nectria radicicola*. Molecular Plant-Microbe Interactions.

[bib0002] Ayllón, M.A., & Vainio, E.J. (2023). Mycoviruses as a part of the global virome: diversity, evolutionary links and lifestyle. In *Advances in Virus Research* (Vol. 115). 10.1016/bs.aivir.2023.02.002. 37173063

[bib0003] Day P.R., Dodds J.A., Elliston J.E., Jaynes R.A., Anagnostakis S.L. (1977). Double-stranded RNA in Endothia parasitica. Phytopathology..

[bib0004] Dálya L.B., Černý M., de la Peña M., Poimala A., Vainio E.J., Hantula J., Botella L. (2024). Diversity and impact of single-stranded RNA viruses in Czech *Heterobasidion* populations. bioRxiv..

[bib0006] Ghabrial S.A., Castón J.R., Jiang D., Nibert M.L., Suzuki N. (2015). 50-plus years of fungal viruses. Virology..

[bib0007] Gonthier P., Garbelotto M. (2011). Amplified fragment length polymorphism and sequence analyses reveal massive gene introgression from the European fungal pathogen *Heterobasidion annosum* into its introduced congener *H. irregulare*. Mol. Ecol..

[bib0008] Hantula, J., Ahtikoski, A., Honkaniemi, J., Huitu, O., Härkönen, M., Kaitera, J., Koivula, M., Korhonen, K., Lindén, A., Lintunen, J., Luoranen, J., Matala, J., Melin, M., Nikula, A., Peltoniemi, M., Piri, T., Räsänen, T., Sorsa, J.A., Strandström, M., Uusivuori, J. & Ylioja, T. (2023). Metsätuhojen kokonaisvaltainen arviointi: mETKOKA-hankkeen loppuraportti. https://urn.fi/URN:ISBN:978-952-380-688-7.

[bib0009] Hantula J., Mäkelä S., Xu P., Brusila V., Nuorteva H., Kashif M., Hyder R., Vainio E.J. (2020). Multiple virus infections on *Heterobasidion* sp. Fungal. Biol..

[bib0010] Hillman, B.I., & Milgroom, M.G. (2021). The ecology and evolution of fungal viruses. In *Studies in Viral Ecology*: 2nd Edition. 10.1002/9781119608370.ch5.

[bib0011] Honkaniemi J., Lehtonen M., Väisänen H., Peltola H. (2017). Effects of wood decay by *Heterobasidion annosum* on the vulnerability of Norway spruce stands to wind damage: a mechanistic modelling approach. Canadian Journal of Forest Research.

[bib0012] Hough B., Steenkamp E., Wingfield B., Read D. (2023). Fungal viruses unveiled: a comprehensive review of mycoviruses. Viruses..

[bib0013] Hyder R., Pennanen T., Hamberg L., Vainio E.J., Piri T., Hantula J. (2013). Two viruses of *Heterobasidion* confer beneficial, cryptic or detrimental effects to their hosts in different situations. Fungal. Ecol..

[bib0014] Ihrmark K., Johannesson H., Stenström E., Stenlid J. (2002). Transmission of double-stranded RNA in *Heterobasidion annosum*. Fungal Genetics and Biology.

[bib0015] Jurvansuu J., Kashif M., Vaario L., Vainio E., Hantula J. (2014). Partitiviruses of a fungal forest pathogen have species-specific quantities of genome segments and transcripts. Virology..

[bib0016] Kashif M., Hyder R., De Vega Perez D., Hantula J., Vainio E.J. (2015). *Heterobasidion* wood decay fungi host diverse and globally distributed viruses related to Helicobasidium mompa partitivirus V70. Virus Res..

[bib0017] Kashif M., Jurvansuu J., Vainio E.J., Hantula J. (2019). Alphapartitiviruses of heterobasidion wood decay fungi affect each other’s transmission and host growth. Front. Cell Infect. Microbiol..

[bib0018] Kondo, H., Botella, L., & Suzuki, N. (2022). Mycovirus diversity and evolution revealed/inferred from recent studies. In *Annual Review of Phytopathology* (Vol. 60). 10.1146/annurev-phyto-021621-122122. 35609970

[bib0019] Korhonen, Kari (1978). Intersterility Groups of *Heterobasidion annosum*. Finnish Forest Research Institute, Vol. 94. p. 25.

[bib42] Korhonen K., Piri T., Johansson M., Stenlid J. (1994).

[bib0020] Kuhn J.H., Botella L., de la Peña M., Vainio E.J., Krupovic M., Lee B.D., Navarro B., Sabanadzovic S., Simmonds P., Turina M. (2024). Ambiviricota, a novel ribovirian phylum for viruses with viroid-like properties. J. Virol..

[bib0021] Müller M.M., Sievänen R., Beuker E., Meesenburg H., Kuuskeri J., Hamberg L., Korhonen K. (2014). Predicting the activity of *Heterobasidion parviporum* on Norway spruce in warming climate from its respiration rate at different temperatures. For. Pathol..

[bib0022] Nevalainen S. (2017). Comparison of damage risks in even-and uneven-aged forestry in Finland. Silva Fennica.

[bib0023] Niemelä, T., & Korhonen, K. (1998). Taxonomy of the genus *Heterobasidion*. In S. Woodward, J. Stenlid, R. Karjalainen, & A. Hutterman (Eds.), *Heterobasidion Annosum*. Biology, Ecology, Impact and Control (p. (p. 589).). CAB International.

[bib0024] Oliva J., Bendz-Hellgren M., Stenlid J. (2011). Spread of *Heterobasidion annosum* s.s. and *Heterobasidion parviporum* in *Picea abies* 15 years after stump inoculation. FEMS Microbiol. Ecol..

[bib0025] Olson Å., Aerts A., Asiegbu F., Belbahri L., Bouzid O., Broberg A., Canbäck B., Coutinho P.M., Cullen D., Dalman K., Deflorio G., van Diepen L.T.A., Dunand C., Duplessis S., Durling M., Gonthier P., Grimwood J., Fossdal C.G., Hansson D., Henrissat B., Hietala A., Himmelstrand K., Hoffmeister D., Högberg N., James T.Y., Karlsson M., Kohler A., Kües U., Lee Y.-H., Lin Y.-C., Lind M., Lindquist E., Lombard V., Lucas S., Lundén K., Morin E., Murat C., Park J., Raffaello T., Rouzé P., Salamov A., Schmutz J., Solheim H., Ståhlberg J., Vélëz H., de Vries R.P., Wiebenga A., Woodward S., Yakovlev I., Garbelotto M., Martin F., Grigoriev I.V., Stenlid J. (2012). Insight into trade-off between wood decay and parasitism from the genome of a fungal forest pathogen. New. Phytol..

[bib0026] Osaki H., Nomura K., Iwanami T., Kanematsu S., Okabe I., Matsumoto N., Sasaki A., Ohtsu Y. (2002). Detection of a double-stranded RNA virus from a strain of the violet root rot fungus *Helicobasidium mompa* Tanaka. Virus. Genes..

[bib0027] Redfern, D.B., & Stenlid, J. (1998). Spore dispersal and infection. In S. Woodward, J. Stenlid, R. Karjalainen, & A. Hüttermann (Eds.), *Heterobasidion annosum*: Biology, ecology, Impact and Control (pp. 105–124). London, England: CAB International.

[bib0005] Roininen E., Vainio E.J., Sutela S., Poimala A., Kashif M., Piri T., Hantula J. (2024). Virus transmission frequencies in the pine root rot pathogen *Heterobasidion annosum*. Virus Res..

[bib0028] Sato Y., Suzuki N. (2023).

[bib0029] Stenlid J. (1985). Population structure of *Heterobasidion annosum* as determined by somatic incompatibility, sexual incompatibility, and isoenzyme patterns. Canadian Journal of Botany.

[bib0030] Sutela S., Piri T., Vainio E.J. (2021). Discovery and community dynamics of novel ssRNA mycoviruses in the conifer pathogen *Heterobasidion parviporum*. Front. Microbiol..

[bib0031] Vainio E.J., Hantula J. (2016). Taxonomy, biogeography and importance of *Heterobasidion* viruses. Virus Res..

[bib0032] Vainio E.J., Jurvansuu J., Hyder R., Kashif M., Piri T., Tuomivirta T., Poimala A., Xu P., Mäkelä S., Nitisa D., Hantula J. (2018). Heterobasidion partitivirus 13 mediates severe growth debilitation and major alterations in the gene expression of a fungal forest pathogen. J. Virol..

[bib0033] Vainio E.J., Jurvansuu J., Streng J., Rajamäki M.L., Hantula J., Valkonen J.P.T. (2015). Diagnosis and discovery of fungal viruses using deep sequencing of small RNAs. Journal of General Virology.

[bib0034] Vainio E.J., Korhonen K., Tuomivirta T.T., Hantula J. (2010). A novel putative partitivirus of the saprotrophic fungus *Heterobasidion ecrustosum* infects pathogenic species of the *Heterobasidion annosum* complex. Fungal. Biol..

[bib0035] Vainio E.J., Müller M.M., Korhonen K., Piri T., Hantula J. (2015). Viruses accumulate in aging infection centers of a fungal forest pathogen. ISME Journal.

[bib0036] Vainio E.J., Pennanen T., Hantula J. (2017). Occurrence of similar mycoviruses in pathogenic, saprotrophic and mycorrhizal fungi inhabiting the same forest stand. FEMS Microbiol. Ecol..

[bib0037] Vainio E.J., Piri T., Hantula J. (2013). Virus community dynamics in the coniferpathogenic fungus *Heterobasidion parviporum* following an artificial introduction of a partitivirus. Microb. Ecol..

[bib0038] van Diepeningen, A.D. (2021). *Biocontrol via mycoviruses: a neglected option for bioprotection?* 10.19103/as.2021.0093.20.

[bib0039] Wang Q., Mu F., Xie J., Cheng J., Fu Y., Jiang D. (2020). A single ssRNA segment encoding RdRp is sufficient for replication, infection, and transmission of ourmia-like virus in fungi. Front. Microbiol..

[bib0040] Woodward, S., Stenlid, J., Karjalainen, R., & Hüttermann, A. (1998). Preface. In S. Woodward, J. Stenlid, R. Karjalainen, & A. Hüttermann (Eds.), *Heterobasidion annosum*: Biology, ecology, Impact and Control (pp. xi–xii). London, England: CAB International.

[bib0041] Yu X., Li B., Fu Y., Jiang D., Ghabrial S.A., Li G., Peng Y., Xie J., Cheng J., Huang J., Yi X. (2010). A geminivirus-related DNA mycovirus that confers hypovirulence to a plant pathogenic fungus. Proc. Natl. Acad. Sci. U.S.A..

