# Supplementary material for: Complex transmission of partiti-, ambi- and ourmiaviruses in the forest pathogen Heterobasidion parviporum
Source: Virus Res. 2024 Oct 9;350:199466. doi: 10.1016/j.virusres.2024.199466 (PMC11736393; doi:10.1016/j.virusres.2024.199466)
Supplement: Supplementary file 1 [file mmc1.docx]

**Supplementary material**

**Table S2.** The isolates included in the RNA-Seq libraries of this study.

| **RNA-seq library** | **SRA accession** | **isolates** |
| --- | --- | --- |
| Kashif | SRR26105418 | KS92 |
|  |  | SB9.3 |
|  |  | SB2011 |
|  |  | LAP3.3.11 |
|  |  | RK5A |
|  |  | + 3 *Heterobasidion parviporum*  + 2 *Heterobasidion annosum* |
|  |  | + 1 *Lactarius tabidus* |
| PhyHet | SRR26105417 | SB9.3 |
|  |  | SB6.26 |
|  |  | + 1 *Heterobasidion annosum* |
|  |  | + 3 *Phytophthora cactorum* |
|  |  |  |

The libraries contained also additional fungal or oomycete isolates not included in the current study (libraries Kashif, and PhyHet.

**Table S3.** Relation of the detected viruses to known viruses, BlastX analyses.

| **Virus** | **Accession number** | **Length of accession (nt)** | **Name of nearest hit BlastX** | **Accession number of BlastX hit** | **Query Cover** | **E- value** | **Percent identity**  **(%)** |
| --- | --- | --- | --- | --- | --- | --- | --- |
| HalV3-pa4 | OR607751 | 5024 | hypothetical protein [Heterobasidion ambi-like virus 3] | UHK02576.1 | 41 % | 0.0 | 97.98 |
| HetOlV1-pa7 | OR343713 | 1977 | RNA-dependent RNA polymerase [Heterobasidion ourmia-like virus 1] | WLV75612.1 | 76 % | 0.0 | 100 |
| HetOlV4-pa2 | OR0883038 | 1978 | RNA-dependent RNA polymerase [Heterobasidion ourmia-like virus 3] | UOX39321.1 | 74 % | 0.0 | 93.90 |
| HetOlV4-pa1 | OR644494 | 2626 | RNA-dependent RNA polymerase [Heterobasidion ourmia-like virus 3] | UOX39321.1 | 73 % | 0.0 | 94.21 |
| HetRV6-pa36 | OR644495 | 1368 | RNA-dependent RNA polymerase [Heterobasidion RNA virus 6] | AHA82553 | 94 % | 0.0 | 95.54 |
| HetRV6-pa36 | OR644496 | 1750 | hypothetical protein QK704_s2gp1 [Heterobasidion RNA virus 6] | YP_010840155 | 63 % | 0.0 | 98.91 |
| HetAlV3-pa3 | OR644497 | 5023 | hypothetical protein [Heterobasidion ambi-like virus 3] | WOH21564.1 | 41 % | 0.0 | 98.99 |
| HetAlV10-pa1 | OR644498 | 4966 | hypothetical protein [Heterobasidion ambi-like virus 12] | WOK44144.1 | 39 % | 0.0 | 98.78 |
| HetAlV12-pa1 | OR644499 | 6159 | hypothetical protein [Heterobasidion ambi-like virus 10] | WOK44142.1 | 32 % | 0.0 | 98.78 |
| HetAlV13-pa1 | OR644500 | 4867 | hypothetical protein [Heterobasidion ambi-like virus 9] | UOX39314.1 | 42 % | 0.0 | 99.42 |
| HetpaFV1-pa1 | OR644501 | 7155 | RNA-dependent RNA polymerase [Lentinula edodes partitivirus 2] | UYO08507.1 | 59 % | 0.0 | 45.80 |
| HetAlV3-pa4 | OR051023 | 2628 | hypothetical protein [Heterobasidion ambi-like virus 3] | UHK02577.1 | 38 % | 0.0 | 99.69 |
| HetAlV3-pa4 | OR051024 | 5024 | hypothetical protein [Heterobasidion ambi-like virus 3] | UHK02577.1 | 38 % | 0.0 | 99.69 |
| HetOlV4-an1 | OR083038 | 1978 | RNA-dependent RNA polymerase [Heterobasidion ourmia-like virus 3] | UOX39321.1 | 74 % | 0.0 | 93.90 |

**Table S4.** Differences in virus transfer frequency between given *H. parviporum* donors.

|  | | | | | |  |  |  |  |  |
| --- | --- | --- | --- | --- | --- | --- | --- | --- | --- | --- |
| **Donors** |  | **Virus** | **Recipient** | **P-value** |  |  |  |  |  |  |
| SB6.26-PV13-OlV4-AlV3 | SB9.3-PV13-OlV4-AlV3 | HetPV13-an1 | RK5A  SB2011 | 0.244  0.339 |  |  |  |  |  |  |
| SB6.26-PV13-OlV4-AlV3 | SB6.26-PV13-15- OlV4-AlV3 | HetPV13-an1 | RK5A  SB2011 | 0.244  0.161 |  |  |  |  |  |  |
| SB6.26-PV13-OlV4-AlV3 | SB6.26-PV13-15- OlV4-AlV3 | HetOlV4-pa2 | RK5A  SB2011 | 0.500  0.500 |  |  |  |  |  |  |
| SB6.26-PV13-OlV4-AlV3 | SB6.26-PV13-15- OlV4-AlV3 | HetAlV3-pa4 | RK5A  SB2011 | 1.000  0.500 |  |  |  |  |  |  |

**Table S5.** P-values of differences in virus transfer frequency between two *H. parviporum* donors infected with two partitiviruses (SB9.3-PV13-15- OlV1-4-AlV3) and a single partitivirus (SB9.3-PV15-OlV1-4-AlV3) in cases of four donor isolates.

| **Virus     Recipient** | **HetPV15-pa1** | **HetOlV1 -pa7** | **HetOlV4 -pa2** | **HetAlV3-pa4** |
| --- | --- | --- | --- | --- |
| RK5A | 0.048* | 0.018* | 0.395 | 0.001** |
| SB2011 | 0.154 | 0.100 | 0.500 | 0.219 |
| LAP3.3.11 | 0.281 | 0.036* | 0.001** | 0.001** |
| KS92 | 1.000 | 0.001** | 0.244 | 0.001** |

 P-value thresholds: ** = ≤ 0.005 , * = ≤ 0.05

1981 aa

HetpaFV1-pa1

7155 nt

RdRp

helicase

Figure S1. Schematic representation of novel Heterobasidion parviporum fusarivirus 1.
